# Colorectal cancer atlas: An integrative resource for genomic and proteomic annotations from colorectal cancer cell lines and tissues

**DOI:** 10.1093/nar/gkv1097

**Published:** 2015-10-22

**Authors:** David Chisanga, Shivakumar Keerthikumar, Mohashin Pathan, Dinuka Ariyaratne, Hina Kalra, Stephanie Boukouris, Nidhi Abraham Mathew, Haidar Al Saffar, Lahiru Gangoda, Ching-Seng Ang, Oliver M. Sieber, John M. Mariadason, Ramanuj Dasgupta, Naveen Chilamkurti, Suresh Mathivanan

**Affiliations:** 1 Department of Computer Science and Information Technology, La Trobe University, Bundoora, Victoria 3086, Australia; 2 Department of Biochemistry and Genetics, La Trobe Institute for Molecular Science, La Trobe University, Melbourne, Victoria 3086, Australia; 3 The Bio21 Molecular Science and Biotechnology Institute, University of Melbourne, Parkville, Victoria 3010, Australia; 4 Walter and Eliza Hall Institute of Medical Research, Parkville, Victoria 3052, Australia; 5 Faculty of Medicine, Dentistry and Health Sciences, Department of Medical Biology, University of Melbourne, Parkville, Victoria 3052, Australia; 6 Olivia Newton John Cancer Research Institute, Melbourne, Victoria 3084, Australia; 7 Ludwig Institute for Cancer Research, Melbourne-Austin Branch, Victoria 3084, Australia; 8 School of Cancer Medicine, La Trobe University, Melbourne, Victoria 3084, Australia; 9 Genome Institute of Singapore, A*STAR, 60 Biopolis Street, Singapore 138672, Singapore

## Abstract

In order to advance our understanding of colorectal cancer (CRC) development and progression, biomedical researchers have generated large amounts of OMICS data from CRC patient samples and representative cell lines. However, these data are deposited in various repositories or in supplementary tables. A database which integrates data from heterogeneous resources and enables analysis of the multidimensional data sets, specifically pertaining to CRC is currently lacking. Here, we have developed Colorectal Cancer Atlas (http://www.colonatlas.org), an integrated web-based resource that catalogues the genomic and proteomic annotations identified in CRC tissues and cell lines. The data catalogued to-date include sequence variations as well as quantitative and non-quantitative protein expression data. The database enables the analysis of these data in the context of signaling pathways, protein–protein interactions, Gene Ontology terms, protein domains and post-translational modifications. Currently, Colorectal Cancer Atlas contains data for >13 711 CRC tissues, >165 CRC cell lines, 62 251 protein identifications, >8.3 million MS/MS spectra, >18 410 genes with sequence variations (404 278 entries) and 351 pathways with sequence variants. Overall, Colorectal Cancer Atlas has been designed to serve as a central resource to facilitate research in CRC.

## INTRODUCTION

Colorectal cancer (CRC) is the third most common form of cancer and has the fourth highest mortality rate in the world (1). In order to advance our understanding of the initiation and progression of this disease, biomedical researchers have performed global analyses of the genome, epigenome, transcriptome, proteome and metabolome of CRC patient samples and representative cell lines (2–5). According to The Cancer Genome Atlas Network (3), APC, TP53, KRAS, PIK3CA, FBXW7, SMAD4, TCF7L2 and NRAS are the most frequently mutated genes in CRC. Identification of these mutations and associated pathways has advanced our understanding of CRC, is enabling the sub-classification of this disease and is unveiling potential new avenues for treatment.

Due to the significant advancements in high-throughput technologies, vast amounts of multidimensional data relevant to the biology of CRC have been generated. To extract meaningful biological insights from these data, researchers previously needed to collate data from a large number of studies. To facilitate this process, a series of databases have been created. For example, cancer gene mutations are currently catalogued in databases including TCGA (3), COSMIC (6), TumorPortal (7), IntOGen (8), Network of Cancer Genes (9) and TSGene (10). These databases provide valuable information of gene variations for a number of tumor types including CRC, however, they are not specifically designed to integrate sequence variations with proteomic data. NetGestal (11) is a web-based framework that allows for integration of OMIC data from multiple species in the context of biological networks (12) and contains data pertaining to human CRC from TCGA. However, there is currently no user-friendly online resource specifically pertaining to CRC which catalogues genomic and proteomic data from literature, databases and TCGA, integrates the sequence variations with protein domain, post-translational modifications and protein–protein interactions.

Here, we describe Colorectal Cancer Atlas (http://www.colonatlas.org), an integrated web-based resource which catalogues genomic and proteomic data from CRC tissues and cell lines. Data catalogued include; quantitative and non-quantitative protein expression, sequence variations, cellular signaling pathways, protein–protein interactions, Gene Ontology terms, protein domains and post-translational modifications (PTMs). Data pertaining to genomic sequence variations and protein expression have been manually curated from the scientific literature and collated from other publicly available databases. Colorectal Cancer Atlas is designed to enable a user to search for a specific mutation in any particular cell line, and search for cell lines with and without specific mutations. Currently, Colorectal Cancer Atlas contains data for >13 711 primary CRC tissues, >165 CRC cell lines, 62 251 protein identifications, >8.3 million MS/MS spectra, >18 410 genes with sequence variations, 404 278 sequence variation entries, 351 pathways with sequence variants, 88 819 PTMs and 253 700 protein–protein interactions (Table 1).

**Table 1.** Colorectal cancer atlas statistics

Protein entries	62 251
MS/MS spectra	8 378 422
Primary tissues	13 711
Cell lines	165
Genes with sequence variants	18 410
Gene sequence variants	404 278
Pathways with genes having sequence variants	351
Pathways with genes having no sequence variants	1657
Cell lines with drug sensitivity	27
PTMs	88 819
PTMs affected by sequence variants	1631
Protein–protein interactions	253 700

## DATABASE ARCHITECTURE AND WEB INTERFACE

Colorectal Cancer Atlas is a web-based application developed using Zope2 (version 2.8.7–1), a python-based web framework. The back end database is MySQL (version 5.0.95), a well-established open source database. The web pages were developed using Hyper Text Markup Language (HTML) in combination with JavaScript for front end functionality, while Python (version 2.4.3), a scripting language was used for database connectivity. JavaScript modules include DataTables (version 1.10.4) for the development of interactive data tables, Data-Driven Documents (D3JS) for the development of interactive protein–protein interaction networks, and Highcharts (version 4.1.6) for the development of interactive heat maps and column charts.

## GENOMIC DATA SETS

Colorectal Cancer Atlas catalogues gene sequence variations present in primary CRC tissues and cell lines which were collated by manual curation of the scientific literature. In addition, the database contains genomic variations identified in CRC cell lines sequenced in-house. For cell lines, where available, the gender and age of the patient is provided, along with the specific cell type, doubling time, culture properties and stage of cancer. This information was obtained from the Cancer Cell Line Encyclopedia (13), ATCC (http://www.atcc.org), COSMIC database and literature. Sequence variation details including the type of sequence variants, putative mutational effects, nucleotide change and amino acid changes are displayed.

## PROTEOMIC DATA SETS

Colorectal Cancer Atlas also catalogues proteomic data collated from multiple resources including the scientific literature (e.g. Zhang *et al*. (5)), Human Protein Atlas (14), Human Proteinpedia (15) and Human Protein Reference Database (16). Experimental techniques used in generating these data included mass spectrometry, Western blotting, immunohistochemistry, confocal microscopy, immunoelectron microscopy and fluorescence-activated cell sorting (FACS). In addition, publicly available label-free quantitative mass spectrometry data for CRC cell lines and tissues were re-analyzed using an in-house proteomics pipeline in order to provide standardized data. The proteomics pipeline involved conversion of raw mass spectrometry data files into the Mascot Generic File Format (MGF) using MsConvert with peak picking (17). The MGF files were then searched using X! Tandem (Sledgehammer edition version 2013.09.01.1) (18) against a target and decoy Human RefSeq protein database. Peptides were further filtered using <5% false discovery rate (FDR) as a cut-off, and quantified using the Normalized Spectral Abundance Factor (NSAF) method (19).

## COLORECTAL CANCER ATLAS PROVIDES AN INTEGRATED VIEW OF MULTIPLE DATA TYPES

**Figure 1.**
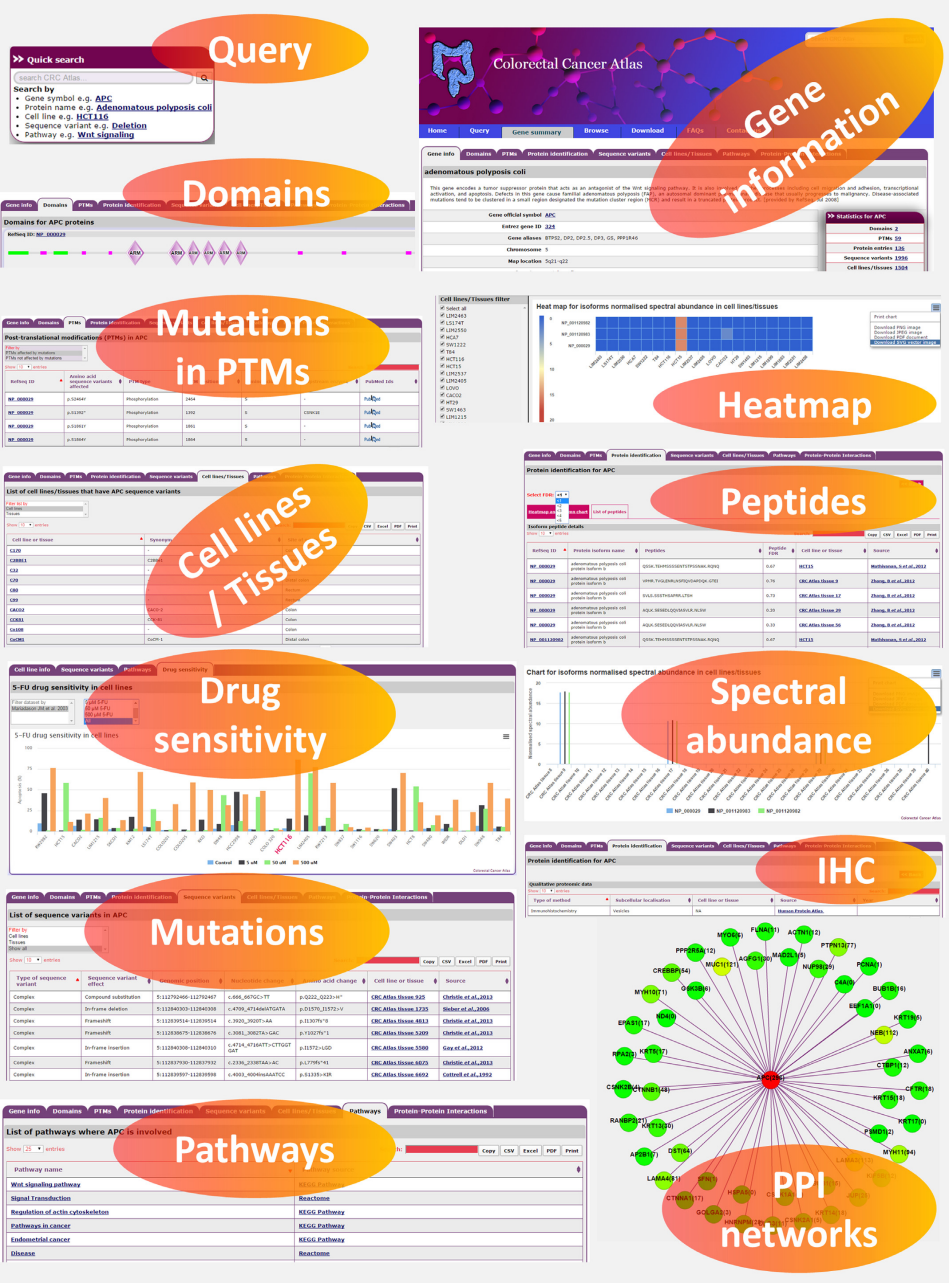
Snapshot of Colorectal Cancer Atlas features. An overview of proteomic and genomic data features for APC gene is displayed. A user can query the database using a gene symbol or a protein name. A gene information page will provide the users with details pertaining to protein domains, post-translational modifications (PTM), reported mutations in cell lines/tissues, quantitative protein expression, pathway, protein–protein interaction (PPI) and cell line drug sensitivity.

Colorectal Cancer Atlas provides an integrated view of the sequence variations and the proteomic data. Mass spectrometry-based quantitative proteomic data are depicted as heat maps and column charts in the respective molecular pages (Figure 1), and users are able to filter the data sets based on the FDR. The database also contains protein expression data generated using immunohistochemistry, Western blotting, FACS, confocal and immunoelectron microscopy. The database also includes protein data derived from various cellular fractions including the nucleus, cytoplasm, membrane, the secretome (20) and exosomes (21) (from ExoCarta (22)).

**Figure 2.**
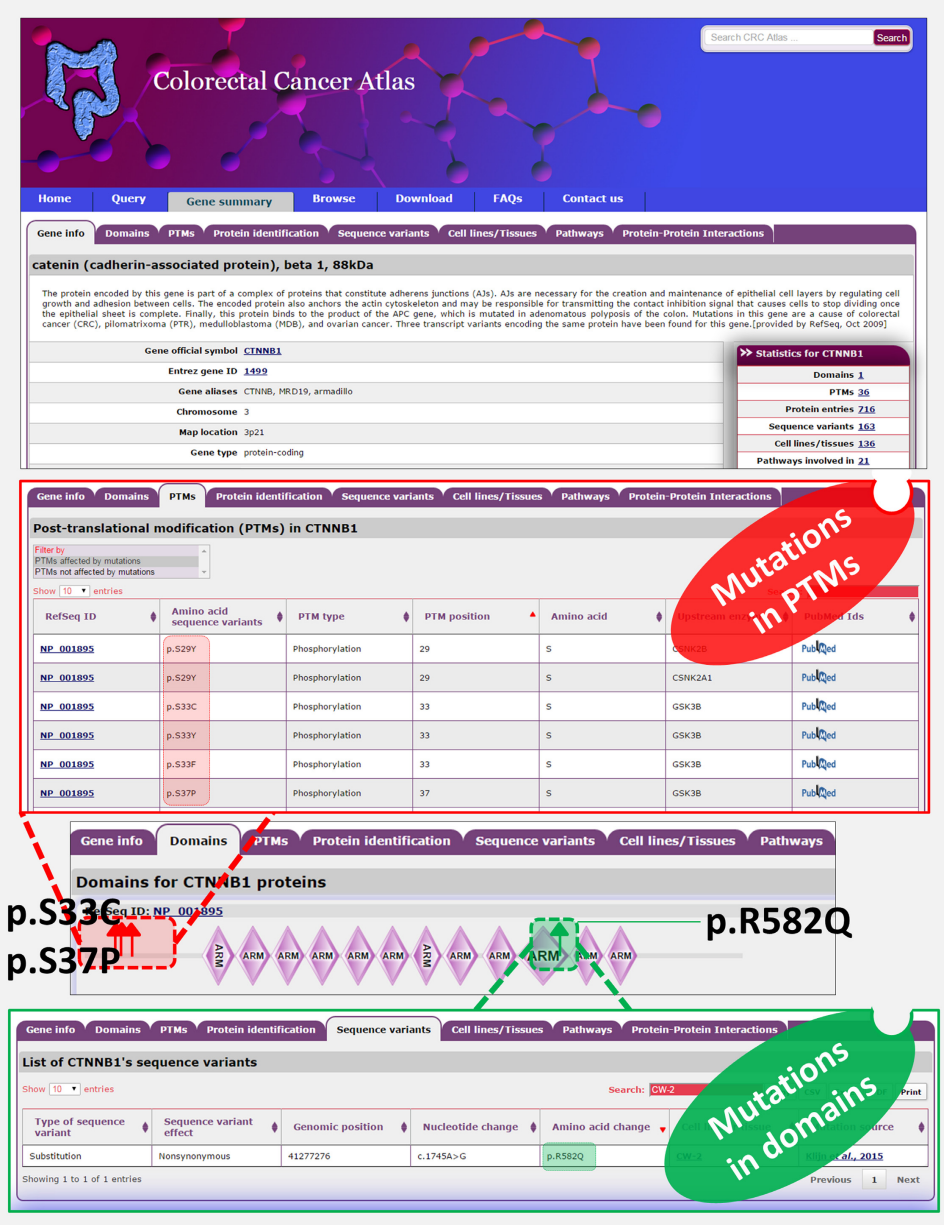
PTMs and domains in β-catenin are affected due to mutation. Snapshot of β-catenin molecular page is displayed. The PTMs affected by mutations can be viewed in the tab PTMs. Mutations in β-catenin at positions important for phosphorylation (S33, S37, T41 and S45) allows for the stabilization of β-catenin and constitutive activation of the Wnt signaling pathway. The upstream kinases responsible for the phosphorylation is also provided along with the literature reference. Likewise, mutations in the armadillo domain can be viewed by correlating the sequence variants and the domain span regions. For example, mutations in the armadillo domain (p.R582) in β-catenin have been described which have been reported to alter the binding of β-catenin to TCF4 (24).

The integration of sequence variants with proteomic data is designed to facilitate the prediction of functional effects of the protein. For each gene, Colorectal Cancer Atlas enables parallel visualization of CRC-associated sequence variants with quantitative protein expression across CRC cell lines and tissues. In addition, PTMs, and protein domains affected by the sequence variation can be visualized (Figure 1), enabling the potential effect of sequence variants on protein function to be easily ascertained. For example, β-catenin mutations in positions S33, S37, T41 and S45 occur in CRC, all of which are critical for phosphorylation (23). Mutations in these serine/threonine residues allow for the stabilization of β-catenin and constitutive activation of the Wnt signaling pathway. Similarly, Colorectal Cancer Atlas displays sequence variations in known protein domains which can provide valuable insight into the putative effect on protein function. For example, mutations in the armadillo domain (R582) in β-catenin have been described which have been reported to alter the binding of β-catenin to TCF4 (24) (Figure 2).

Colorectal Cancer Atlas also provides a graphical representation of known protein interactions (obtained from BioGrid (25) and Human Protein Resource Database (16)), where each protein is depicted as a node with a specific colour and intensity corresponding to the number of sequence variants in the encoding gene (Figure 1). Furthermore, Colorectal Cancer Atlas integrates biological pathways with gene sequence variants. Biological Pathways were obtained from Reactome (26), KEGG (27), Cell map and HumanCyc. For example, as shown in Figure 1, sequence variants in APC are implicated in dysregulation of the Wnt signaling pathway and actin cytoskeletal remodeling. Finally, Colorectal Cancer Atlas contains data on 5-flurouracil (5-FU) drug sensitivity for CRC cell lines curated from the literature (studies using at least three CRC cell lines (28)). Users can view the sensitivity profile of a cell line of interest relative to other CRC cells.

## ACCESSING COLORECTAL CANCER ATLAS

Users can search Colorectal Cancer Atlas through the home, query or browse pages (Supplementary Figure S1). In addition, the website features a navigation menu and a search box at the top of the page. The database can be queried by gene symbol, Entrez Gene ID, protein name, cell line name or pathway. The browse page provides users with the option to access the database by categorized lists of genes, sequence variations, cell lines and techniques. The browse page allows the users to search for sequence variations in genes of interest and displays them in interactive color-coded table format. The gene information page includes gene details, associated GO terms, sequence variations (displayed in an interactive table), domain details, PTMs, a protein data page leading to experimental techniques and quantitative data with an interactive heat map, a column chart for spectral abundance and a list of detected peptides. Other information includes a list of cell lines and tissues that contain sequence variants in a given gene, a list of pathways in which the gene is involved, and an interactive protein–protein interaction network for the protein encoded by the gene. The cell line page provides details of the cell line, an interactive table of gene sequence variants identified in the cell line, an interactive table of dysregulated pathways and 5-FU drug sensitivity profile. Data curated in Colorectal Cancer Atlas are available as tab-delimited files and is free for download to all users. Using the custom database option, the tab delimited data can also be uploaded into FunRich (29), a functional enrichment analysis tool to identify classes of genes/proteins that are overrepresented in a specific category.

## FUTURE DIRECTIONS

Colorectal Cancer Atlas will be continuously updated with more studies as they become available and additional features. Studies currently being curated include Wnt signaling activity determined by the TOPFLASH assay, and genomic and proteomic data generated from patient derived xenografts.
